# Medical Education and Artificial Intelligence: Some Suggestions

**DOI:** 10.31662/jmaj.2025-0335

**Published:** 2025-11-21

**Authors:** Shigeki Matsubara

**Affiliations:** 1 Department of Obstetrics and Gynecology, Jichi Medical University, Tochigi, Japan; 2 Department of Obstetrics and Gynecology, Koga Red Cross Hospital, Koga, Ibaraki, Japan; 3 Medical Examination Center, Ibaraki Western Medical Center, Chikusei, Ibaraki, Japan

**Keywords:** artificial intelligence, ChatGPT, education, medical students, quiz

## Dear Editors,

I believe that providing factual data is fundamental to medicine, including medical education. The study by Amano et al. ^(1)^ is significant: they showed that some medical students positively, whereas others negatively, evaluated artificial intelligence (AI) use in physical and/or medical reasoning. This contradicted my initial assumption that many students would be surprised by the ability of AI to create thoughtful answers efficiently. With this in mind, I would like to offer three suggestions.

First, I have a humble concern about the generalizability of the results. I realized the reason my expectation was unusual: “One reason for students’ dissatisfaction was that students found it time-consuming to verify ChatGPT’s responses. Many believed it would be faster to consult authoritative sources themselves” because Chat Generative Pre-trained Transformer (ChatGPT) often made mistakes. Some students expressed a similar opinion regarding medical writing. Their evaluations appeared to reflect “perceived real-world effectiveness” in this specific physiology quiz. I would respectfully ask the authors: how about providing a different type of quiz, in which students tend to make more mistakes and AI is more likely to give accurate answers? Moreover, this study used ChatGPT-3.5―an older model. If a different task and ChatGPT-4 had been used, evaluations might have changed considerably.

Second, the authors offered a novel educational angle: that AI’s shortcomings (as cited above) could serve as teaching tools. They proposed using AI as a “spot-the-mistake” (*machigai-sagashi*) exercise. Because AI often generates incorrect responses, students are prompted to identify errors―a process familiar from medical examinations. This will also be useful when reviewing AI-edited articles students may write. Such vigilance is considered mandatory ^(2), (3)^.

Third, I would like to ask the authors about their stance on AI in medical reasoning and writing. Although beyond the scope of their study, educators inevitably hold personal positions on what they teach. For instance, I favor AI use in clinical decision-making but believe writing should be strictly regulated ^(3), (4)^. Educators need not impose views on students, but clarifying their stance within such an article may promote productive engagement. Readers might benefit from understanding the authors’ broader perspective. Such two-way reflection could enrich discussion on medical education and education itself.

I agree with the authors’ conclusion: “AI can support learning but cannot yet fully replace traditional educational methods.” This holds true not only in medical education but also in medicine, and society in general. I am not a medical education specialist but a seasoned clinician-researcher involved in teaching. The voice of a non-specialist may sometimes prove helpful.

## Article Information

### Author Contributions

Shigeki Matsubara: Manuscript writing.

### Conflicts of Interest

None

### Data Availability

Data sharing is not applicable to this article because no new data were created or analyzed in this study.
